# ARID3A Dysregulation Drives Colon Cancer Progression and Enhances Responsiveness to Aspirin

**DOI:** 10.1111/jcmm.71038

**Published:** 2026-01-28

**Authors:** Jiade Li, Muhan Li, Quanfu Li, Yungaowa Wu, Yifan Shen, Yanping Li, Mingshuo Zhang, Guangyou Wang, Yuanyuan Zhu

**Affiliations:** ^1^ Department of Neurobiology School of Basic Medical, Harbin Medical University Harbin Heilongjiang P.R. China; ^2^ Department of Biotherapy Center Harbin Medical University Cancer Hospital Harbin P.R. China; ^3^ Department of Pathology School of Basic Medical, Harbin Medical University Harbin Heilongjiang P.R. China; ^4^ Department of Medical Oncology Ordos Central Hospital Ordos Inner Mongolia P.R. China

**Keywords:** ARID family, ARID3A, aspirin, colon cancer, macrophage

## Abstract

The AT‐Rich Interaction Domain (ARID) family plays critical roles in malignancies. Although numerous members have been shown to influence cancer processes, there is a lack of a general understanding of the ARID family in colon cancer. To address this gap, we used bioinformatic technologies to investigate the role of the ARID family as a whole and to identify the crucial member. Subsequently, cell growth assays, transwell assays, and animal models were employed to validate the key member's effect on colon cancer growth and metastasis. Furthermore, bioinformatics and immunohistochemistry were utilised to explore the potential mechanisms and evaluate the efficacy of a targeted intervention strategy. Our results showed that the ARID family was upregulated in colon cancer, with ARID3A being the main component that promoted colon cancer development. Specifically, ARID3A enhanced colon cancer cell proliferation, migration, and invasion both in vivo and in vitro. Mechanistically, this promotional effect could be associated with ARID3A promoting PGE2 synthesis and triggering macrophage infiltration. Notably, aspirin treatment reduced the PGE2 level, which significantly inhibited the malignant behaviour of ARID3A‐overexpressing cells. In conclusion, ARID3A was a key member of the ARID family in the development of colon cancer. ARID3A was an underlying biomarker for aspirin administration.

## 1 | Introduction

The AT‐Rich interaction domain (ARID) family is a superfamily whose members possess a DNA binding domain enabling them to bind to specific DNA, modify chromatin structure, and regulate gene transcription [1, 2]. The aberrant expression or dysfunction of ARID family members has been linked to cancer [1]. Currently, there are now 15 ARID family members, which can either promote or repress cancer. Different members may interact synergistically or antagonistically. For example, both ARID3B and ARID3A enhanced the expression of stemness‐related genes in ovarian cancer cells. Moreover, ARID3A and ARID3B promoted each other's expression and regulated nearly identical gene sets, confirming their collaborative nature [3]. ARID4A and ARID4B belong to the same subgroup, but ARID4A acts as a tumour suppressor in prostate and breast cancer [4], whereas ARID4B is upregulated in hepatocellular carcinoma (HCC) and is associated with a poor prognosis [5]. Some research has revealed the role of the ARID family in cancer. Sun et al. investigated the full ARID family landscape in HCC [6]. Although multiple members were elevated and indicated a poor prognosis, multivariate analysis identified only ARID3A, ARID5B, and ARID1A as independent prognostic indicators, and the three‐gene signature displayed good prediction accuracy for HCC prognosis [6, 7]. The ARID family also plays a role in tumour immunity. Studies have confirmed that ARID3A overexpression can promote B2 cell proliferation, indicating its positive regulatory role in B2 cell expansion. Mechanistically, ARID3A influences B cell survival and differentiation by regulating the expression of genes related to B cell development, such as Myc and Bhlhe41. During B1a cell development, ARID3A enhances BCR signalling by reducing the expression of Siglec‐G and CD72 (negative regulators of BCR signalling), thereby supporting the positive selection of B1a cells [8]. ARID3A is highly expressed in Treg cells, particularly in activated Treg cells, where its expression level is significantly elevated. Mice with Treg cell‐specific knockout of ARID3A exhibit reduced Treg cell numbers and diminished survival capacity. Mechanistic studies reveal that ARID3A enhances IL‐2 signalling by upregulating CD25 expression, thereby maintaining Treg cell stability and suppressive function [9]. Overexpression of ARID3A markedly promotes Th17 cell differentiation, suggesting that ARID3A is a positive regulator of Th17 cell differentiation. Mechanistic investigations demonstrate that ARID3A directly binds to the promoter region of the RORγt gene, enhancing its transcriptional activity, which implies that ARID3A drives Th17 cell differentiation by upregulating RORγt expression [10]. Research has verified that ARID3A is essential for sustaining CD8^+^T cell function. ARID3A can directly bind to the promoter region of the T‐bet gene, promoting its transcription, suggesting that ARID3A sustains the effector function of CD8^+^T cells by upregulating T‐bet expression. Additionally, research has found that in ARID3A‐deficient CD8^+^T cells, the expression of exhaustion‐related genes (Pdcd1, Havcr2) is further elevated, indicating that ARID3A may also maintain cellular function by suppressing excessive exhaustion [11]. Zhu et al. discovered that ARID family members are novel biomarkers for identifying the subgroup of cancers that benefit most from immune checkpoint inhibitor (ICI) treatment [12]. Examining ARID family members in different types of malignancies can improve prognosis prediction; however, their prognostic utility varies [1]. Lu et al. conducted a systematic study of the ARID family and its association with stemness, tumour microenvironment, and prognosis in digestive cancer, and discovered significant heterogeneity in the expression or function of ARID family members, implying the need to study the ARID family members individually in various cancers [13].

ARID1A deficiency in colorectal cancer (CRC) promotes cancer growth by impairing SWI/SNF regulation of enhancers bound by dominant transcription factors (TFs) that are required for development and differentiation [14]. Previous research has shown that ARID3A enhances the development and chemosensitivity of CRC by modulating AURKA and AKR1C3 [15, 16]. However, no study has comprehensively and systematically examined ARID family members in CRC. It remains unclear which members play crucial roles and how they play them. This study investigated the expression of ARID family in colon cancer. We identified the putative ARID family member's function and mechanism and proposed viable interventions. This study will provide us with a more comprehensive understanding of the ARID family in colon cancer, as well as an effective intervention strategy for colon cancer patients with aberrant ARID family profiles.

## 2 | Methods

### 2.1 | Bioinformation Technique

In this study, RNA seq data from ARID family members were enriched using single sample gene set enrichment analysis (ssGSEA). The obtained enrichment scores were utilized to analyse the relative activity differences of ARID genes in cancer and normal tissue samples, allowing us to quantify functional performance differences between cancer types. The expression of 15 ARID family members was compared in colon cancer and normal tissues using GEPIA. The immune cells infiltrating in colon cancer tissues were examined utilising GEPIA. Using the TCGA database, we examined the relationship between ARID3A and prognosis and clinical indicators. The level of ARID3A protein was determined using mass spectrometry data from the Human Protein Atlas (THPA) database.

### 2.2 | Human Tissues and Immunohistochemistry (IHC)

IHC tests were used to assess the expression of ARID3A and CD68 in cancer tissues from 59 colon cancer patients. Briefly, formaldehyde‐fixed paraffin embedded (FFPE) tissue sections were dewaxed and rehydrated. After blocking endogenous peroxidase activity and retrieving the antigen, the sections were treated overnight at 4°C with anti‐ARID3A antibody (Proteintech: No. 14068–1‐AP) or anti‐CD68 antibody (Abcam: No. ab213363), followed by a 30‐min incubation with the secondary antibody at 37°C. DAB staining was used to demonstrate a positive outcome, and the sections were counterstained with haematoxylin. Two pathologists independently evaluated the staining results. ARID3A expression was represented by an IHC score, which was calculated by multiplying the staining area score by the staining intensity score, while CD68‐marked cells were counted under a microscope and represented by the number of positive cells per high power (HP). The Harbin Medical University Institutional Ethics Committee approved this work. Participants gave written informed consent prior to their involvement in this study.

### 2.3 | Cell Culture

The cell lines employed in this investigation were HCT116, SW1116, and HEK293T. All cells were grown in RPMI‐1640 media with 10% foetal bovine serum (FBS) (Invitrogen, Carlsbad, CA, USA), 100 U/mL streptomycin, and 100 mg/mL penicillin. Cells were kept in a 37°C incubator with 5% CO_2_.To examine the effects of aspirin in vitro, 2 mM was added to the growth medium. Every 3 months, mycoplasma contamination was evaluated by PCR. The test cells were cultured in double‐antibody‐free medium for 7 days. After that, 100 μL of supernatant was collected. The sample was heated in a 95°C water bath for 5 min. Then, 10 μL of StrataClean resin was added to the tube, vigorously mixed using a vortex mixer, and centrifuged for 10 s. The supernatant was aspirated for the PCR reaction, and appropriate agarose gel electrophoresis markers were selected for electrophoresis. After electrophoresis, the experimental results were analysed using a gel imaging system. The primers: F‐ACACCATGGGAGCTGGTAAT, R‐CTTCTTCGACTTCCAGACCCAAGGCAT.

### 2.4 | Plasmids and Transfection

ARID3A shRNA was constructed using known sequences and then put into the lentiviral expression vector PLKO. The plasmid for ARID3A overexpression was created by cloning ARID3A into the PLVX‐PURO lentiviral vector. Lentiviral particles were generated by transfecting these lentiviral vectors into 293 T cells along with the packaging system plasmids PS (Addgene) and PM (Addgene). A 10‐cm culture dish with 5 × 106 293 T cells was transfected with 12 μg lentiviral vector, 9 μg PS, and 3 μg PM using the Lipo3000 (Thermo Scientific, Waltham, MA, USA). The supernatant from 293 T cells was collected and filtered, and HCT116 and SW1116 cells were transduced with lentiviral particles. ARID3A overexpression or knockdown was verified using a Western Blot. The accompanying primers refer to the previous article [15].

### 2.5 | Western Blot

Cell samples were lysed on ice for 30 min with RIPA lysate supplemented with protease inhibitor (Beyotime), then centrifuged, the supernatant collected, and the protein content measured using a BCA kit (Beyotime). This work used anti‐ARID3A antibody (Proteintech: No. 14068‐1‐AP) and ACTIN (ZSGB‐BIO, Beijing, China: No. TA‐09). A protein sample was plated onto a 12% SDS‐PAGE gel. After electrophoresis, proteins were transferred to a PVDF membrane and treated with the primary antibody at 4°C overnight. Following that, the appropriate secondary antibody was added and incubated for 1 h at room temperature. Colour rendering was used to visualise protein bands, which were then shot and preserved.

### 2.6 | Cell Growth Curve

Cells (4 × 10^4^ per well) were seeded into 6‐well plates. After incubating for 24, 48, or 72 h, the cells in each well were resuspended in 1 mL PBS. Growth curves were generated according to the results from automatic cell counting using the Accuri C6 Cytometer (BD Biosciences).

### 2.7 | Transwell Assay

The migration and invasion tests were performed using a 24‐well transwell chamber system (Corning Incorporated, Corning, New York, USA). In brief, 5 × 10^4^ cells were resuspended in 400 μL of serum‐free media and seeded into the upper chamber with or without Matrigel. The lower chamber received 700 μL of medium containing 20% serum. After 24 h of incubation, the chambers were discoloured. Migrant and invasive cells were imaged and counted.

### 2.8 | Animal Models

We acquired 4‐week‐old male nude mice from Vital River Laboratory (Beijing, China) and injected 1 × 10^6^ cells subcutaneously into the leg pits. Once palpable tumours were identified, their size was assessed every 3 days. The mice were euthanized 4 weeks later. The tumours were excised, measured, and weighed. In the metastatic tumour model, 1 × 10^6^ cells resuspended in 50 μL PBS were injected into nude mice via the tail vein. The mice were euthanized after 4 weeks. The lung tissue was removed and sectioned, and the number of cancer nodules was determined using a microscope. To evaluate the effects of aspirin, mice were given either aspirin (100 mg/kg) or PBS intragastrically every day till the experiment ended. The Harbin Medical University Committee approved the study, and all experiments adhered to the Guide for the Care and Use of Laboratory Animals.

### 2.9 | RNA Sequence

Total RNA was extracted utilising Trizol reagent (Thermofisher) in accordance with the manufacturer's protocol, and the quantity and purity of RNA were assessed using the Bioanalyzer 2100 and RNA 6000 Nano LabChip Kit (Agilent). Following the purification of mRNA utilising Dynabeads Oligo (dT) (Thermo Fisher) and subsequent fragmentation into short segments, the RNA fragments underwent reverse transcription to synthesise cDNA using SuperScript II Reverse Transcriptase (Invitrogen), after which the results were amplified via PCR. High throughput sequencing (PE150) was conducted using the Illumina Novaseq 6000 sequencer (LC‐Bio Technology CO. Ltd., Hangzhou, China).

### 2.10 | ELISA Assay

PGE2 generated by cells was identified using an ELISA kit (Elabscience Biotechnology Co. Ltd., Wuhan, Hubei, China, No. E‐EL‐0034c), as stated in the manufacturer's handbook. Each well received 50 μL of serially diluted reference sample or supernatant. 50 μL of biotinylated detection antibody was added to each well and incubated for 45 min at 37°C. HRP conjugate, substrate reagent, and Stop Solution were then added to the wells in order, and the absorbance was measured at 450 nm. The result was calculated using the standard curve.

### 2.11 | Statistical Analysis

The data were presented as mean ± SD. The student's *t*‐test was used to compare the means of two groups. The relationship between ARID3A and clinical indicators was explored using the chi‐square test. The proliferation was analysed using one‐way ANOVA. *p*‐values < 0.05 indicated statistical significance. *p < 0.05, **p < 0.01, ***p < 0.001, ***p < 0.0001, ns = not significant.

## 3 | Results

### 3.1 | ARID3A Is the Most Important Member of ARID Family Involved in CRC Development

**FIGURE 1:**
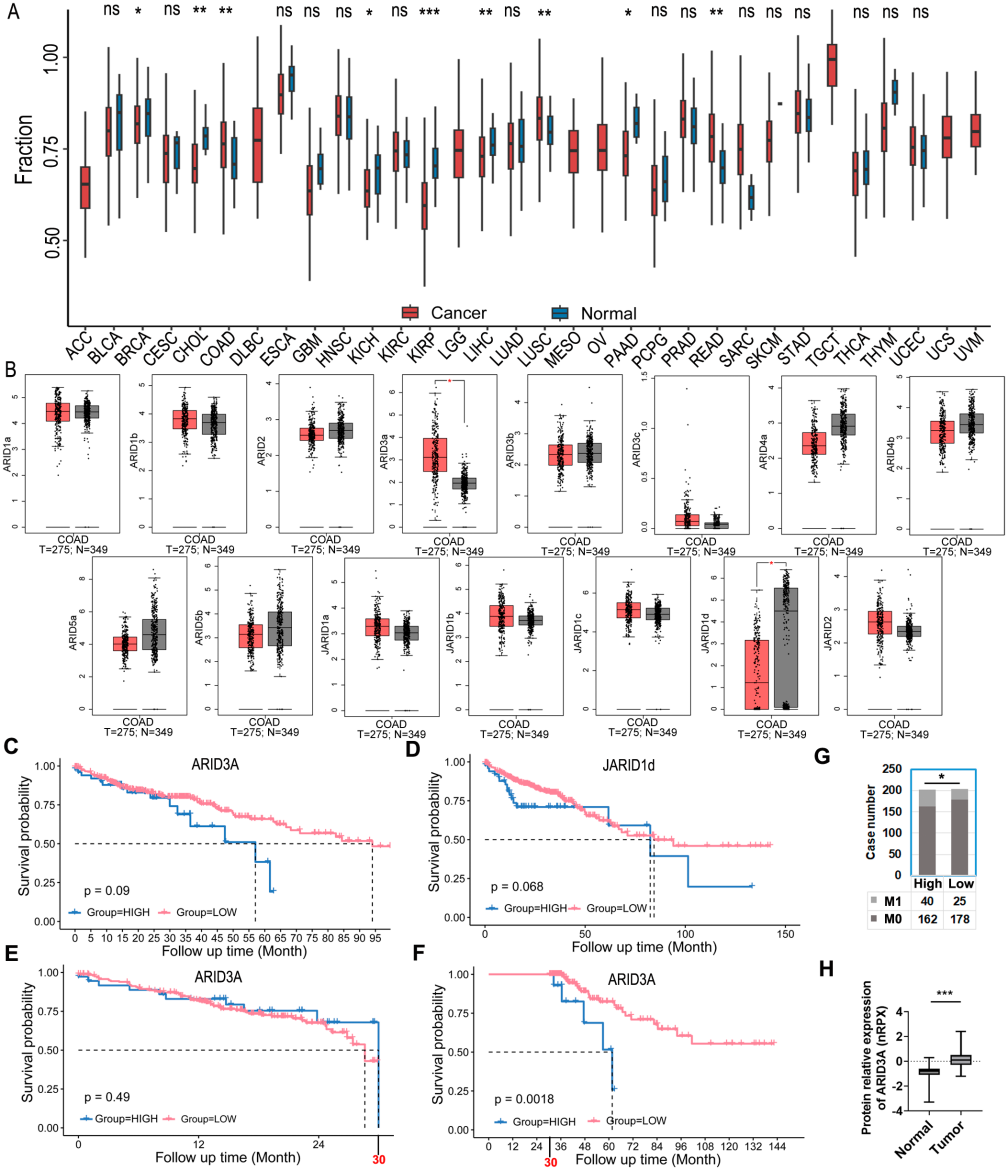
ARID3A is the principal member of the ARID family that facilitates the development of colon cancer. (A) Scores from ssGSEA were utilised to assess the activity differences of the ARID family between cancerous and normal tissues. *p < 0.05, **p < 0.01, ***p < 0.001. (B) GEPIA was utilised to examine the expression of each member of the ARID family in colon cancer. *p < 0.05. (C, D) The OS analysis was conducted based on the levels of ARID3A or JARID1d. (E, F) The impact of ARID3A on the short‐term and long‐term prognosis of colon cancer patients was evaluated by taking the 30‐month limit. (G) The column diagram illustrated the correlation between ARID3A levels and metastases in colon cancer patients. *p < 0.05. (H) The ARID3A protein was studied based on the THPA database. ***p < 0.001.

We used the TCGA database to evaluate the expression of the ARID family in 32 types of common malignancies and matching normal tissues, taking 15 members into account. The results showed that the ARID family was considerably elevated in three types of malignancies and downregulated in six types of cancers, indicating that the ARID family plays an important role in cancer development (Figure 1A). The level of the ARID family as a whole was higher in colon cancer than in normal tissues, but subsequent analysis revealed that only two members altered significantly: ARID3A was upregulated and JARID1d was downregulated (Figure 1B). ARID3A and JARID1d had no effect on colon cancer patients' prognosis (Figure 1C,D). However, when we evaluated the influence of ARID3A on colon cancer patient prognosis in a segmented fashion, we discovered that the level of ARID3A had no effect on the short‐term prognosis (Figure 1E), while a high level of ARID3A was a negative factor for the long‐term prognosis (Figure 1F). We then examined the relationship between ARID3A and clinical indicators, and the findings revealed that high levels of ARID3A were related to distant metastases in patients (Table 1). Individuals with high ARID3A expression had considerably more distant metastases than individuals with low ARID3A expression (Figure 1G). Furthermore, ARID3A overexpression in colon cancer was validated at the protein level using mass spectrometry data from the Human Protein Atlas (Figure 1H).

**TABLE 1:** The relationship between ARID3A mRNA and clinical parameters.

Clinical parameter	ARID3A mRNA expression		*p*
	High	Low	
	(*N* = 202)	(*N* = 203)	
Age			
< 65	79 (39.1%)	66 (32.5%)	> 0.05
≥ 65	123 (60.9%)	137 (67.5%)	> 0.05
Gender			
Female	91 (45.0%)	102 (50.2%)	> 0.05
Male	111 (55.0%)	101 (49.8%)	> 0.05
Metastasis			
M0	162 (80.2%)	178 (87.7%)	< 0.05
M1	40 (19.8%)	25 (12.3%)	< 0.05
Lymph node			
N0	118 (58.4%)	124 (61.1%)	> 0.05
N1‐2	84 (41.6%)	70 (38.9%)	> 0.05
Tumour			
T1 and T2	38 (18.8%)	40 (19.7%)	> 0.05
T3 and T4	164 (81.2%)	163 (80.3%)	> 0.05
Stage			
I and II	111 (55.0%)	122 (60.1%)	> 0.05
III and IV	91 (45.0%)	81 (39.9%)	> 0.05

These findings suggested that ARID3A is a crucial member of the ARID family involved in colon cancer. The overexpression of ARID3A in colon cancer may contribute to the progression of the disease and indicate a poor prognosis for patients.

### 3.2 | ARID3A Promotes the Proliferation and Metastasis of Colon Cancer Cells

**FIGURE 2:**
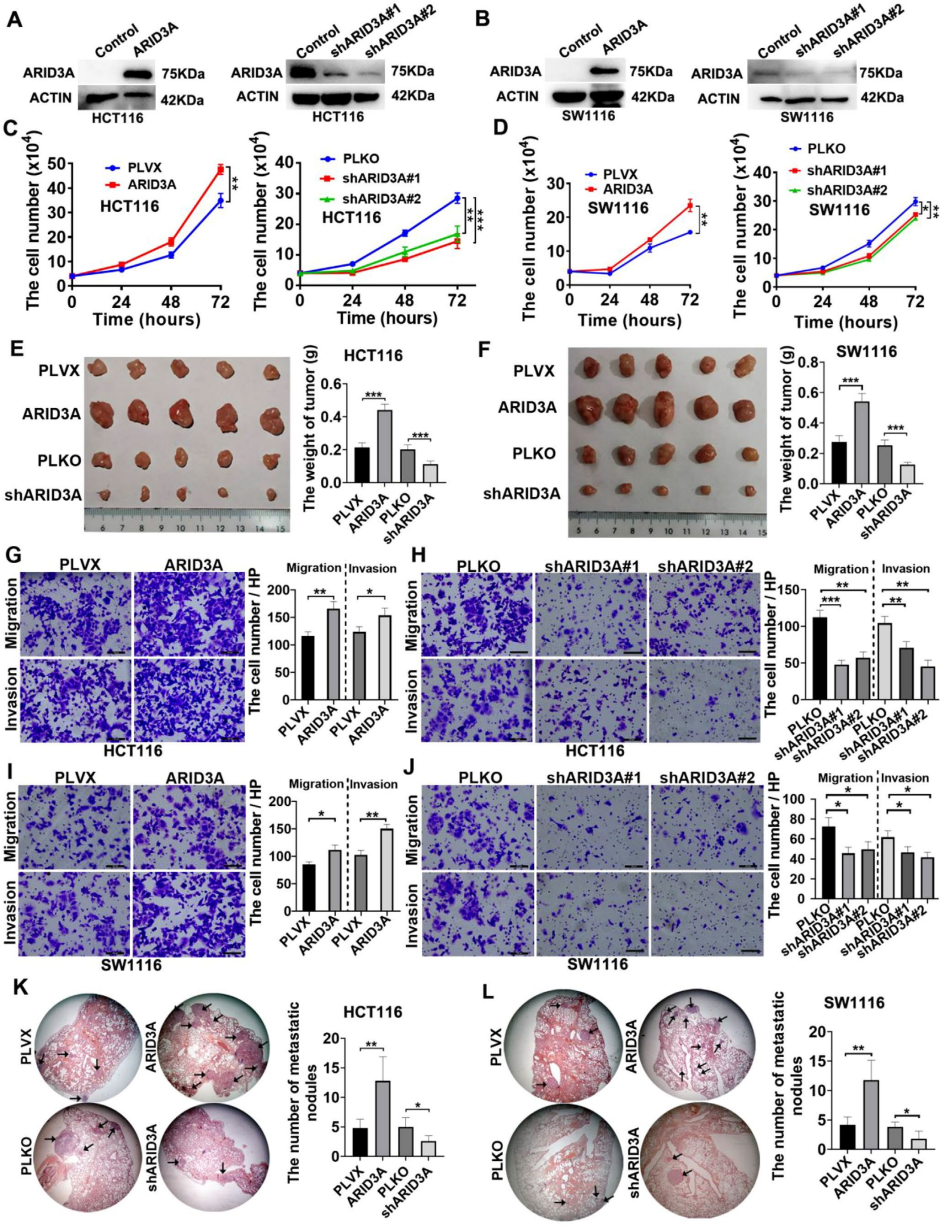
ARID3A facilitates the progression of colon cancer cells. (A, B) The overexpression and knockdown were validated by a western blot test. (C, D) The proliferative capacity of colon cancer cells was assessed utilising a cell growth curve. *p < 0.05, **p < 0.01, ***p < 0.001. (E, F) Subcutaneous tumour formation tests were employed to assess the impact of ARID3A on cancer cell proliferation in vivo. ***p < 0.001. (G, H, I, J) The impact of ARID3A on the migration and invasion of colon cancer cells was assessed using a transwell assay. Scale bars, 100 μm. *p < 0.05, **p < 0.01, ***p < 0.001. (K, L) The metastatic capacity of colon cancer cells in vivo was evaluated using a lung metastatic tumour model. *p < 0.05, **p < 0.01.

To investigate the function of ARID3A in colon cancer, we constructed colon cancer cells with ARID3A overexpression or knockdown (Figure 2A,B). The cell growth curve revealed that ARID3A increased the proliferative capacity of HCT116 and SW1116 cells, but ARID3A knockdown reduced their proliferative ability (Figure 2C,D). The results of subcutaneous tumour development demonstrated that ARID3A overexpression increased the growth of HCT116 and SW1116, whereas ARID3A knockdown greatly prevented the growth of two colon cancer cell lines in vivo (Figure 2E,F). Transwell experiment verified ARID3A's beneficial influence on colon cancer cells' migratory and invasive capacities, which increased when ARID3A was overexpressed but decreased when ARID3A was knocked down (Figure 2G–J). When colon cancer cells were injected into mice's tail veins, more ARID3A overexpressing cells metastasized and colonised in their lungs, whereas fewer ARID3A knock‐down cells metastasized and colonised in their lungs (Figure 2K,L). Thus, ARID3A increased the proliferative and metastatic abilities of colon cancer cells in vitro and in vivo.

### 3.3 | ARID3A Contributing to CRC May Be Related With Macrophage Infiltration

**FIGURE 3:**
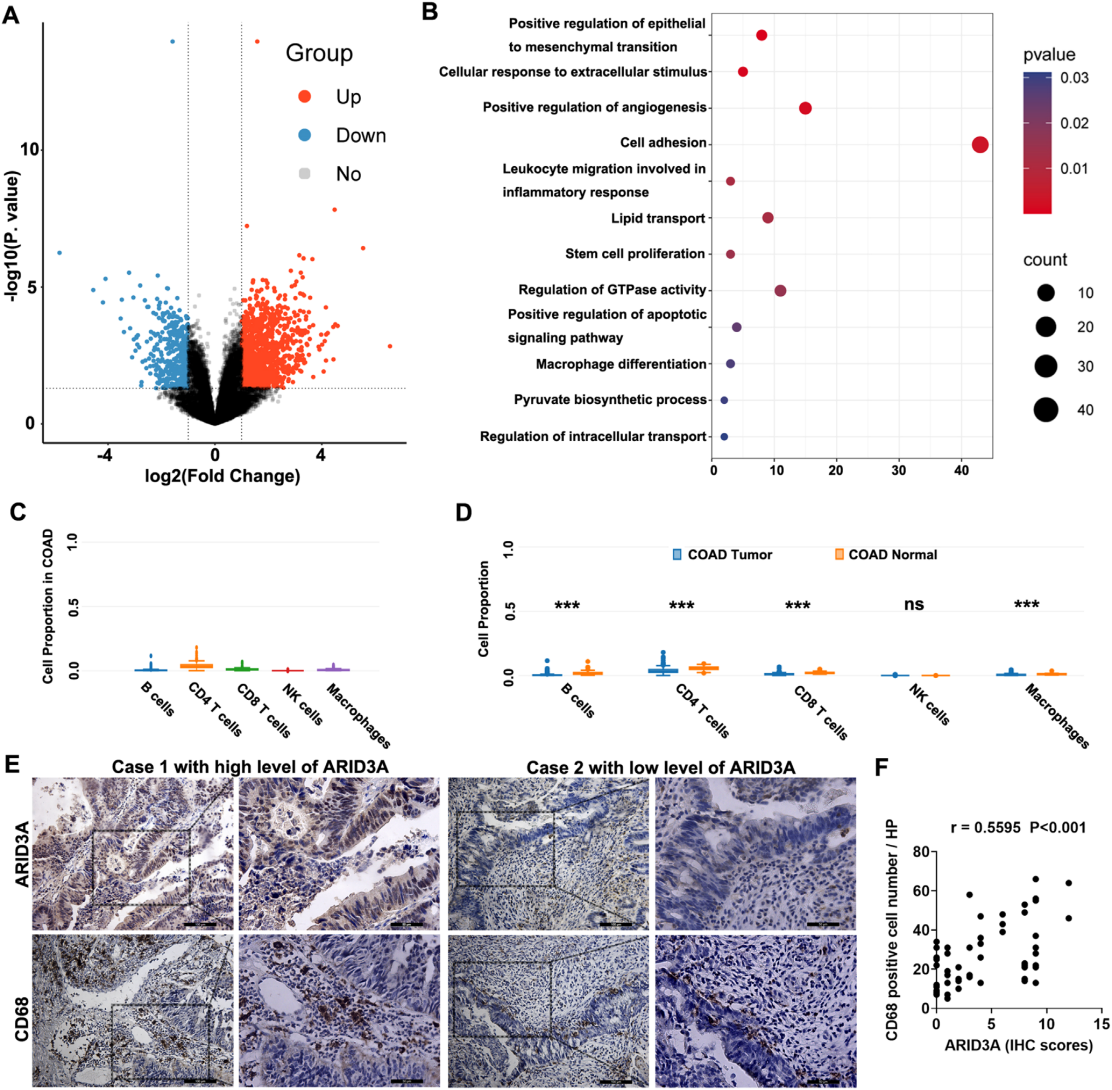
ARID3A may promote macrophage infiltration in colon cancer. (A) RNA sequencing revealed the differentially expressed genes in cells overexpressing ARID3A relative to control cells. (B) Gene Ontology functional enrichment analysis was conducted on the differentially expressed genes. (C, D) Immune cells in colon cancer were examined utilising the GEPIA database. ***p < 0.001. (E) The sample immunohistochemical pictures from two colon cancer cases demonstrated the expression of ARID3A and CD68. Scale bars, 100 μm. The box in the overview indicates the magnified area. Scale bars, 50 μm. (F) The relationship between ARID3A expression and macrophage infiltration was examined based on IHC results.

To establish the mechanism by which ARID3A works in CRC cancer growth, we used RNA sequencing to evaluate the differentially expressed genes in SW1116 cells following ARID3A overexpression. The results revealed that ARID3A overexpression increased the expression of 2082 genes while decreasing the expression of 1192 genes by more than 1.5 times in SW1116 cells (Figure 3A). The GO functional enrichment analysis revealed that ARID3A had a considerable impact on inflammation and immune‐associated genes, such as leukocyte migration and macrophage differentiation (Figure 3B). We used the GEPIA database to investigate the kinds of infiltrating immune cells in colon cancer. The results showed that five types of immune cells may be identified in colon cancer tissues, with NK cells being exceedingly rare, while three types of lymphocytes and macrophages were plentiful (Figure 3C). In comparison to normal tissues, NK cells did not alter much; three kinds of lymphocytes were decreased, and macrophages were increased in colon cancer (Figure 3D). Thus, macrophages may be key immune cells that contribute to colon cancer. Next, we examined the expression of ARID3A and the relationship between ARID3A expression and macrophage infiltration in colon cancer tissues. The findings revealed that colon cancer tissues with high levels of ARID3A included more macrophages labelled with CD68. The typical photos were shown in Figure 3E. ARID3A expression linked strongly with macrophage infiltration (Figure 3F). The findings suggested that ARID3A may enhance the development of colon cancer by promoting macrophage infiltration.

### 3.4 | Aspirin Inhibits CRC Progression by Blocking PGE2 Production

**FIGURE 4:**
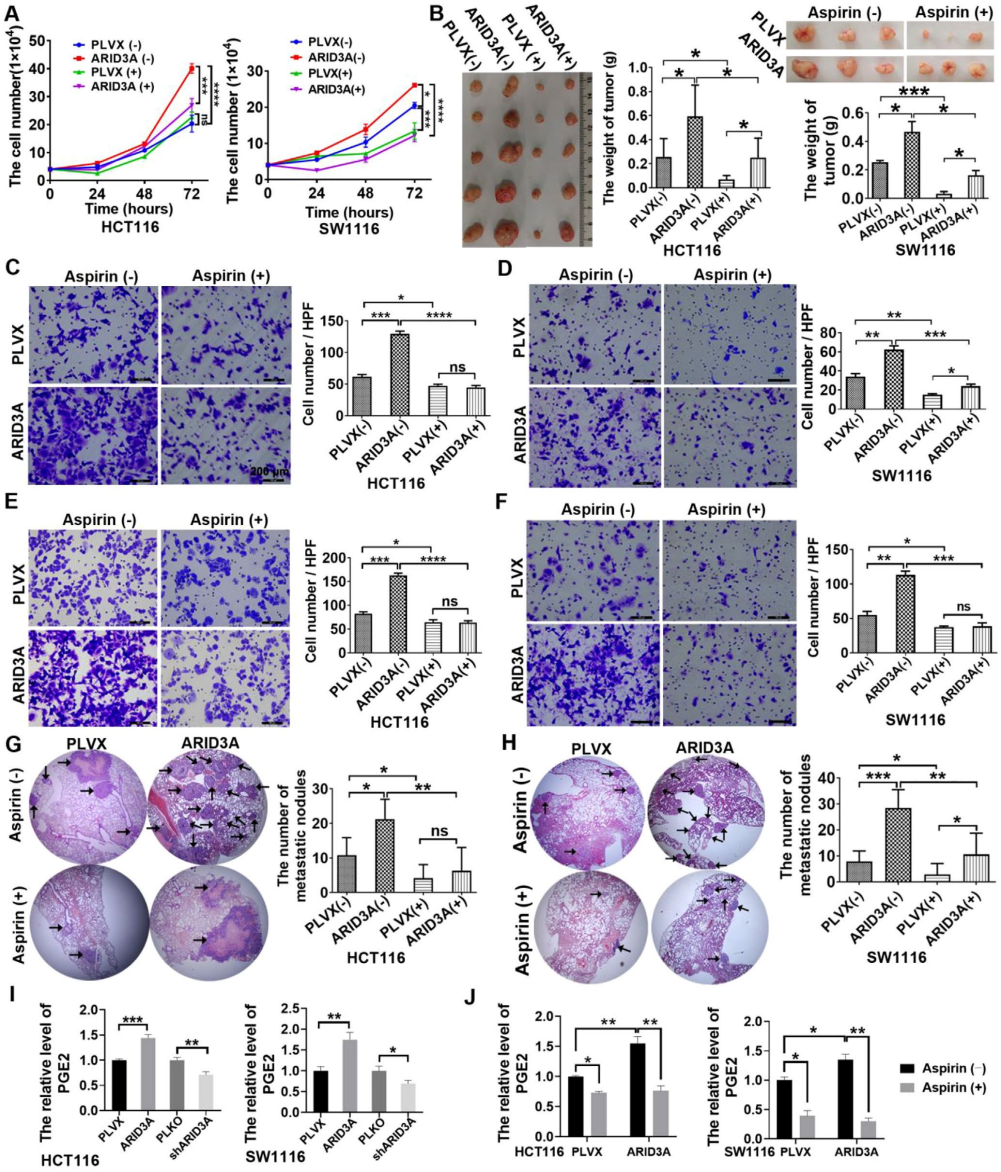
Aspirin exerts a superior inhibitory effect on ARID3A‐overexpressing cells by suppressing PGE2. (A) The in vitro proliferative capacity was evaluated by a cell growth curve. *p < 0.05, **p < 0.01, ***p < 0.001, ****p < 0.0001. (B) The in vivo proliferative capacity was evaluated using a subcutaneous xenograft model, which indicated that ARID3A exhibits a stronger response to aspirin. The tumour weight was represented via a column graphic. *p < 0.05, ***p < 0.001. (C, D, E, F) The transwell assay was employed to assess the migratory and invasive capabilities of colon cancer cells. The statistical analysis was shown in a column diagram reflecting the number of transmembrane cells per high power field. Scale bars, 100 μm. *p < 0.05, **p < 0.01, ***p < 0.001, ****p < 0.0001. (G, H) A xenograft model, utilising tail vein injection, was employed to evaluate the metastatic potential of colon cancer cells in vivo. The column graphic illustrated the quantity of metastatic nodules in the lung. *p < 0.05, **p < 0.01, ***p < 0.001. (I, J) Relative expression level of PGE2 (mean ± SEM, *n* = 3 technical replicates) as determined by ELISA. *p < 0.05, **p < 0.01, ***p < 0.001.

It is well understood that the inflammatory process plays an essential role in the onset and progression of CRC [17]. Multiple studies have found that nonsteroidal anti‐inflammatory medications (NSAIDs) can lower the risk of CRC onset and development by decreasing inflammation. Because ARID3A can promote tumour growth by promoting macrophage infiltration, we hypothesised that NSAIDs would be more effective in treating CRC cells with high ARID3A levels. The cell growth curve demonstrated that aspirin suppressed the proliferation of colon cancer cells, with a more pronounced inhibitory impact observed in ARID3A overexpressed cells compared to control cells (Figure 4A). The in vivo assay verified aspirin's inhibitory effect on colon cancer cells; however, no advantage was observed in ARID3A overexpressed cells (Figure 4B). The migration assay (Figure 4C,D) and invasion assay (Figure 4E,F) demonstrated that aspirin can impede the migratory and invasive capabilities of CRC cells, with a more pronounced inhibitory impact observed in colon cancer cells exhibiting ARID3A overexpression compared to normal cells. The in vivo assay revealed a greater number of metastatic nodules and a more pronounced inhibitory impact of aspirin in ARID3A overexpressed cells (Figure 4G,H). We demonstrated that aspirin decreased the progression of colon cancer cells, particularly those with ARID3A overexpression.

Aspirin's primary mechanism in combating cancer is the reduction of PGE2. The ELISA assay demonstrated that ARID3A overexpression enhances, whereas knockdown diminishes, PGE2 synthesis in colon cancer cells (Figure 4I). The administration of aspirin can diminish the synthesis of PGE2, particularly in cells with overexpression of ARID3A (Figure 4J). The data suggested that ARID3A may enhance macrophage infiltration by stimulating the synthesis of the inflammatory mediator PGE2. Aspirin may exert a superior inhibitory impact on ARID3A‐overexpressing cells via suppressing PGE2.

## 4 Discussion

ARID3A is a transcription factor that may be implicated in cancer proliferation, metastasis, and chemoresistance by modulating the production of coding or non‐coding RNA [18, 19]. This study identified ARID3A as a pivotal member of the ARID family involved in the progression of colon cancer. Furthermore, ARID3A may facilitate the progression of colon cancer by eliciting inflammation, as evidenced by increased macrophage infiltration in tissues with elevated ARID3A levels. The pro‐tumorigenic action of ARID3A has been documented in many cancers. ARID3A enhanced HCC cell survival and metastasis by interacting with CEP131, subsequently transcriptionally activating KDM3A, which is involved in stemness regulation [20]. Liao et al. discovered that ARID3B facilitates the stem‐like phenotype and immune evasion of CRC cells by enhancing the transcription of Notch target genes, intestinal stem cell (ISC) genes, and programmed death‐ligand 1 (PD‐L1) [21]. Mao et al. discovered that ARID3A promotes the growth and chemoresistance of pancreatic cancer by suppressing PTEN transcription, thus resulting in a decrease in ferroptosis [22]. In non‐small cell lung cancer (NSCLC), ARID3A and ARID3B may facilitate cancer progression by upregulating the non‐coding RNAs MALAT1 and NORAD [23]. Based on the aforementioned research, drugs specifically inhibiting ARID3A function can be developed. Currently, relevant platforms are available to provide critical guidance for screening candidate compounds targeting ARID3A or its downstream pathways [24, 25]. In addition, certain studies have suggested that elevated levels of ARID3A correlate with a good prognosis in CRC patients [26, 27]. The downregulation of ARID3A and ARID3B mitigated the senescence programme and facilitated malignant transformation, a critical mechanism in Aflatoxin B1‐induced liver cancer [28]. E2F1 can enhance the expression of ARID3A or facilitate the binding of ARID3A to E2F‐targeted genes, thereby inhibiting the proliferation and metastasis of osteosarcoma cells. ARID3A, a primary target of miR‐125b, was downregulated due to elevated levels of miR‐125b in acute megakaryoblastic leukaemia (AMKL), thereby obstructing dual megakaryocytic/erythroid differentiation. Conversely, reinstating ARID3A expression alleviated the impediment of megakaryocytic differentiation in AMKL patient‐derived xenografts [29]. In osteosarcoma, miR‐361‐3p was identified as an inhibitor of proliferation, migration, and invasion through the negative regulation of ARID3A [30]. Currently, the conclusions on the role of ARID3A in malignancies remain inconsistent, even within the same cancer type.

Our prior research has validated the enhancing effect of ARID3A on the proliferation and metastatic potential of colon cancer in vitro [15]. This research additionally validated this impact in vivo. Furthermore, we confirmed the important significance of ARID3A among ARID family members in colon cancer. This study indicates that elevated levels of ARID3A significantly reduce the long‐term overall survival (exceeding 30 months) of colon cancer patients. This may be due to the fact that most evaluated individuals underwent chemotherapy. Our prior research demonstrated that elevated levels of ARID3A enhanced the chemosensitivity of colon cancer cells [16], however, after 30 months, the benefit of ARID3A in chemotherapy diminishes. Macrophages are pivotal immune cells within the tumour microenvironment of colon cancer and are generally recognised for their essential role in cancer progression [31]. Macrophages are categorised into two subsets: M1 and M2. Research indicates that the majority of infiltrating macrophages have M2‐like characteristics in cancer [32]. This study revealed a positive correlation between ARID3A levels and macrophage infiltration. LncRNA HMMR‐AS1 has been found to upregulate ARID3A expression by competitively binding to miR‐147a, thereby promoting M2 macrophage polarisation and facilitating the progression of HCC [33]. Consequently, we hypothesised that the induction of macrophage infiltration and subtype conversion may be a mechanism through which ARID3A facilitates the progression of colon cancer. In the development of cancer, PGE2 has both autocrine and paracrine effects [34]. PGE2 is a crucial cytokine for the recruitment of macrophages into tumour tissue [35] and promotes M2 polarisation [36]. Additionally, PGE2 can bind to receptors and enhance the malignant phenotype of cancer cells. Despite the unknown mechanism, we discovered that ARID3A elevated PGE2 production. Aspirin can decrease PGE2 synthesis and the proliferation, migration, and invasion of colon cancer cells, especially in cells with overexpressed ARID3A. Given the close association between ARID3A, PGE2, and macrophages, the development of drugs that specifically inhibit ARID3A function or block its interaction with the PGE2 synthesis pathway has become feasible. The molecular properties and toxicity of these drugs need to be predicted and evaluated, and we can also utilise various models for comprehensive assessment [37, 38]. Such drugs can not only attenuate the malignant characteristics of tumour cells but also improve the tumour microenvironment by regulating macrophage infiltration and polarisation.

## 5 Conclusions

In conclusion, ARID3A is a pivotal factor that is upregulated in colon cancer and enhances the malignant phenotype of cancer cells, correlating with the overall survival of colon cancer patients. This may be associated with the increased synthesis of PGE2 in cells overexpressing ARID3A and the stimulation of macrophage infiltration and polarisation. The administration of aspirin in cells overexpressing ARID3A may show enhanced therapeutic efficacy. Therefore, further investigation into the optimal dosage and treatment duration of aspirin for colon carcinoma patients with high ARID3A expression is justified. This strategy holds the potential to provide an economical and effective treatment solution for cancer patients.

## Author Contributions

Y.Z. and G.W. designed experiments; J.L. and G.Y.W. wrote the manuscript; J.L., M.L., and Y.S. performed most of the experiments; M.Z. analysed the data; Q.L. and Y.W. performed experiments and assisted with data analysis; J.L. and Y.P.L. prepared the figures; J.L. prepared the tables; Y.Z. and G.W. supervised the project.

## Funding

This work was supported by the Natural Science Foundation of Heilongjiang Province (LH2023H010 to Y.Z.), Public Hospital Research Joint Fund Science and Technology Project (2024GLLH1311 to Y.W.), the Open Project Program of Key Laboratory of Preservation of Human Genetic Resources and Disease Control in China (Harbin Medical University), Ministry of Education (LPHGRD 2023‐006 to Y.Z.), and the Science foundation of Haiyan (JJQN2019‐06 to J.L).

## Conflicts of Interest

The authors declare no conflicts of interest.

## Data Availability

The data that support the findings of this study are available on request from the corresponding author. The data are not publicly available due to privacy or ethical restrictions.
